# Health Tract, No. 277

**Published:** 1867-03

**Authors:** 


HEALTH TRACT, No. 277.

### THE TOMATOE

Is, perhaps, liked more than any other vegetable.

It is remarkably productive.

It is uncommonly nutritious.

It is indisputably healthful.

It is equally advantageous to the system raw or cooked; whether cold or hot; whether eaten alone, or with salt, or sugar, or vinegar.

Its proper season is until the fore part of Autumn, but if shortly before that, the vines are hung up in a well ventilated cellar not too warm or too dry, the tomatoe will continue to ripen until christmas. This important fact ought to be made known to the widest extent.

The reason of the unusual healthfulness of the "Love Apple" of olden time, when in our easy recollection it was cultivated only as an ornament for the garden and the mantle-piece, is worthy of being explained.

Chemical Physiology has demonstrated that all acids have the effect to clear the bile out of the system by stimulating the liver to increased activity. It is this excess of bile in the blood in the Spring of the year which makes it impure or as some call it, "bad blood," or thick blood, and which our grand-dames used to seek to "thin" or purify, by drenching us with sassafras tea or choking us with powdered brimstone in molasses. Hence it is that by an unappeasable instinct, nature yearns for something sour in the Spring, and we are impatient for the early fruits, and berries and first spinnach, not because of the spinnach itself, but because it is known to be eaten with vinegar, and it is the acid that is craved. So also do persons crave something sour when they are getting bilious; or are recovering from a bilious attack, or are simply a little feverish, which means that a bilious attack is impending, and which acids taken freely, will avert with great certainty. It is the pleasant acid in the tomatoe which makes it healthful as a blood purifier; so pleasant it is, that large quantities can be taken without oppressing the system.

But in another important direction is the friendly tomatoe peculiarly promotive of a healthful condition of the body; the seed, like those of the white mustard, pass through the alimentary canal unchanged and tend to promote that daily regularity of the system, without which good health is not possible of continuance for forty-eight hours ahead. These seed act mechanically on the mucous membrane of the alimentary apparatus, causing it to cast off and wash out those waste matters, the retention of which is the prolific cause of not only the ordinary diseases but of some of the most dangerous and speedily fatal maladies. If women, children, sedentary men and invalids, and persons in poor health generally, could be induced during the warm weather to live almost wholly on coarse bread, samp, hominy, wheaten grits, with fruits, berries and tomatoes, an incalculatable amount of Summer and Autumnal diseases would be avoided.

